# A Scoping Review of Household Factors Contributing to Dietary Quality and Food Security in Low-Income Households with School-Age Children in the United States

**DOI:** 10.1016/j.advnut.2023.05.006

**Published:** 2023-05-13

**Authors:** Heather A. Eicher-Miller, Lisa Graves, Bethany McGowan, Barbara J. Mayfield, Blake A. Connolly, Wanda Stevens, Angela Abbott

**Affiliations:** 1 Department of Nutrition Science, Purdue University, West Lafayette, IN, USA; 2 Libraries and School of Information Studies, Purdue University, West Lafayette, IN, USA; 3 Nutrition Communicator, LLC, Delphi, IN, USA; 4 College of Health and Human Sciences, Purdue Extension, Purdue University, West Lafayette, IN, USA

**Keywords:** food insecurity, food security, low-income, dietary quality, dietary selection, dietary behavior, family meal planning, food purchasing, household, children

## Abstract

Low-income and food-insecure households are at risk of poor dietary quality and even more severe food insecurity. Especially in childhood, consuming a nutritionally adequate diet is an essential driver of health, growth, and development. Household-level factors can present challenges to support the nutritional needs of low-income and food-insecure household members. The aim of this scoping review is to identify the contributing household factors to dietary quality and food security in US households of school-aged children 5 to 19 years and synthesize the evidence around emergent themes for application to future interventions. The scoping review was conducted following the Preferred Reporting Items for Systematic Reviews and Meta-Analysis Protocols Extension for Scoping Reviews using search terms addressing food insecurity, low income, and dietary behaviors in the database PubMed. Screening by 3 independent reviewers of the title, abstract, and full study phases identified 44 studies. The 5 themes around which the studies grouped were: parental behaviors, child/adolescent behaviors, food procurement behaviors, food preparation behaviors, and household environment factors. Most studies were cross-sectional (n = 41, 93%) and focused on parental behaviors (n = 31, 70%), followed by food preparation and procurement behaviors. The themes identified were interrelated and suggest that incorporating education on parent and child behaviors that influence food procurement and preparation, along with strengthening organization and planning in the household environment, may hold promise to improve dietary quality and food security among food-insecure and low-income households. The findings can be used to inform future nutrition education interventions aimed at improving dietary quality and food security in households with school-aged children.

**Statement of significance**

Household factors of parental and child/adolescent behaviors, food procurement and preparation behaviors, and the household environment are contributors to dietary quality and food security in low-income households for children in the United States. Addressing these factors holds promise for interventions to improve dietary quality and food security.

## Introduction

About 10.2% of households, or 13.5 million households, within the United States experienced food insecurity at some time throughout 2021 [1]. Food insecurity refers to a situation where households experience uncertainty regarding having sufficient food or the inability to obtain enough food to meet the needs of all family members due to limited money or resources [1]. Food-insecure households are at risk of reduced dietary quality when compared with households with food security [2,3]. Dietary quality as defined by the Dietary Guidelines for Americans (DGA) [4,5] may be lower in food-insecure compared with food-secure households due to irregular dietary patterns, such as skipping meals and eating less or more of certain components than are intended or required for health [[6], [7], [8]]. Low dietary quality may be a contributor to the link of food insecurity with increased prevalence of chronic disease in adults [[9], [10], [11]].

Specifically for children, consuming a nutritionally adequate diet is an important driver in physical and mental health, growth, development, and wellbeing [5]. Childhood is a life stage when specific amounts and types of nutrients are necessary at critical times to achieve full genetic potential [5] and where decreased macronutrients important to emotional regulation and anxiety of food insecurity may link to mental health outcomes [12]. School-aged children 5 to 19 years interface with the National School Lunch and School Breakfast Programs and begin to practice autonomy in their dietary behaviors. Once established, these behaviors may continue into adulthood [5,13,14]. Food insecurity during childhood may be associated with the consumption of a nutritionally inadequate diet, low dietary quality, and poor health outcomes compared with food security [3,[15], [16], [17], [18]]. For example, school-aged children who are food insecure are more likely to have asthma, poor health, and are nearly 3 times more likely to have iron deficiency anemia than food-secure children [16,19,20].

Insufficient money to purchase adequate food is a significant risk factor for food insecurity [1,21] and poor dietary quality [[22], [23]]. Regardless of individuals having the necessary knowledge and skills, a limited budget may result in opting for food of more economical value instead of food with the most nutritional value. Households with lower incomes purchase foods of lower nutritional quality when compared to households with higher incomes [24,25]. Along with financial restraints, dietary choices can be influenced by additional household factors, such as food accessibility and preferences, eating behaviors, parental and child nutrition knowledge, parental modeling, and psychosocial factors [26,27]. A comprehensive look at the body of evidence of household factors contributing to food security and dietary quality in low-income households with school-aged children 5 to 19 years in the United States and their synthesis or organization around themes has not been previously completed [28]. A scoping review integrating this research evidence may inform future nutrition education programming and interventions to adults or households of school-aged children by identifying, organizing, and showing relationships among recognized family priorities and challenges in obtaining high dietary quality and food security within the limited resource context, as well as identifying any existing gaps in knowledge. The social ecological model serves as a framework for this study and an application for the results. Using the social ecological model, individuals are influenced by interpersonal relationships and their household settings, then the organizations they interact with, and the communities they are situated in and further, the policy levels. The model is also used as the framework for the DGA, showing how these layers of influence can intersect to shape dietary quality. Knowledge of the influence exerted by individuals, interpersonal relationships, and household settings in dietary quality and food security is critical to inform successful interventions directed through the outer levels of the social ecological model, ie, organizational, community, and policy levels. These levels are where federal food assistance and nutrition education programming intervene. Therefore, the purpose of this scoping review was to map current evidence of household factors influencing dietary quality and food security within low-income US households with school-aged children around major themes and to consider relationships of the themes and their factors. The results can be applied to inform future nutrition education interventions and programming.

## Methods

The Preferred Reporting Items for Systematic Reviews and Meta-Analysis Protocols Extension for Scoping Reviews was utilized to conduct this scoping review [29]. Search strategies were developed collectively through discussion among the researchers involved in the project. The following search terms were used: (food insecurity OR food security OR low-income OR poverty) AND United States AND (dietary quality OR dietary behavior OR dietary selection OR dietary attitudes OR family meal planning OR food purchasing). The online database, PubMed, was identified to have a scope fitting of the aspects of focus in the study (food insecurity, dietary quality, household factors) and was used to search for studies written in English and published within the last 10 years between 2012 and 2022, and the search was completed from February 2022 to March 2022 and updated in January 2023 to include all studies published in 2022.

Studies were included in this review if they focused on food security and/or dietary quality of low-income households in the United States and identified factors/behaviors/attitudes/barriers affecting food security and/or dietary quality in these households. Dietary quality for the purposes of the review was inclusive of aspects of dietary behaviors, variety, food components, food group, or nutrient intakes. Studies were excluded if they focused on households that did not include children or did not include school-aged children aged 5 to 19 years, low-income, or food-insecure households.

**FIGURE 1:**
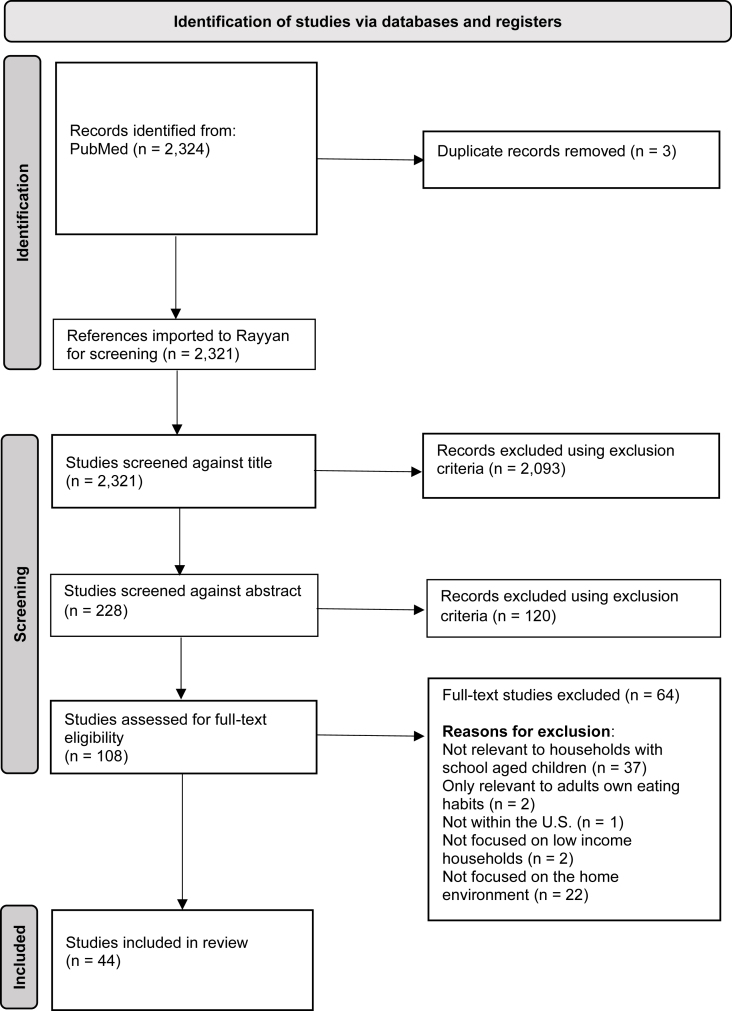
Preferred Reporting Items for Systematic Reviews and Meta-Analysis Protocols flow diagram of 44 studies addressing household factors of dietary quality and food security in US households of school-aged children from PubMed, years 2012 to December 2022.

The PubMed (NCBI) search identified 2324 studies. The search results were exported to EndNote and Rayyan software, which was used to remove duplicates (*n* = 3). Studies were reviewed at 3 stages: title, abstract, and full text. Each stage entailed evaluation by 2 independent reviewers, and a third independent reviewer served as a tie-breaker when there was a discrepancy in the decisions. First, titles and abstracts were screened based on eligibility criteria. Studies were either marked as “included,” “excluded,” or “maybe” by the reviewers. Any studies that were marked as “maybe” were treated as “included.” Next, the remaining chosen studies were screened by reading the full text using the same process as the screening for previous stages. One hundred and eight studies were selected for full-text evaluation. Forty-four studies were included in the scoping review (Figure 1).

**FIGURE 2:**
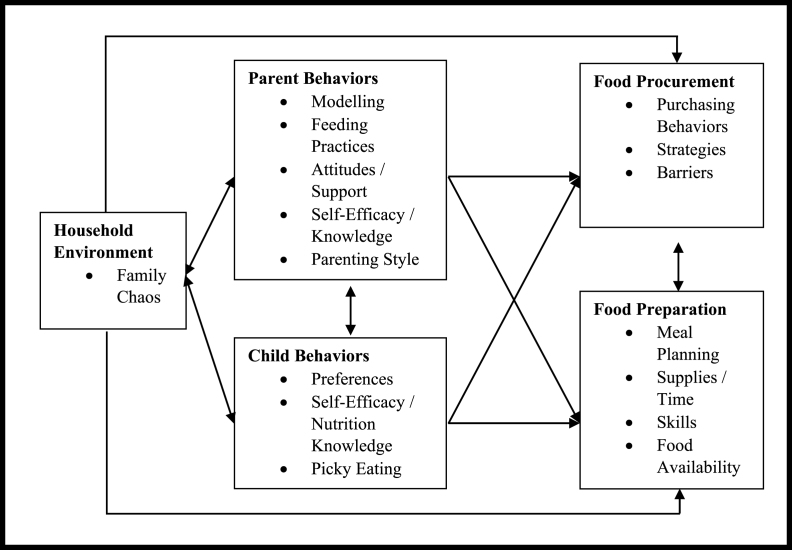
Conceptualized relationships of themes identified among 44 studies addressing household factors of dietary quality and food security in US households of school-aged children from PubMed, years 2012 to December 2022.

The study year, title, author(s), aims, study design, studied population, and main findings were extracted and presented in Table 1 and were used to identify household factors that each study addressed and then to organize the studies to themes. Contributing factors to dietary quality and food insecurity in low-income households were organized into the following 5 themes that emerged from the studies represented in Table 2: *1*) parental behaviors, *2*) adolescent/child behaviors, *3*) food procurement behaviors, *4*) food preparation behaviors, and *5*) household environment. After reading the studies and synthesizing the evidence, the factors organized to themes were mapped to show relationships in Figure 2, which were conceptualized within the framework of the social ecological model, placing the individual and their dietary quality and food security at the center, with interpersonal and household levels of influence shaping the behavior of individuals and encompassing the household factors addressed in this review. Beyond these levels, organizational, community, and policy levels extend with further relevance on the individual, interpersonal, and household levels. The organizational, community, and policy levels are outside of the scope of this review as they provide levels where the findings may be applied.

**TABLE 1:** Dates of publication, titles, authors, aims, study design, population of focus and main findings for 44 studies addressing household factors of dietary quality and food security in US households of school-aged children from PubMed, years 2012 to December 2022.

Year	Title	Authors	Aim	Study Design	Population	Outcomes/Findings
2020	Associations between parent and child physical activity and eating behaviors in a diverse sample: an ecological momentary assessment study	Wirthlin R; Linde JA; Trofholz A; Tate A; Loth K; Berge JM	Examine the association between parent modeling of physical activity and dietary intake and child dietary intake and physical activity	Observational, quantitative, cross-sectional	(n = 150) Families w/ children (5–7 y)	Parent modeling of dietary intake was significantly linked to child dietary intake. Parent modeling of fruit and vegetable intake was associated with increased fruit and vegetable intake in children. Parent modeling of energy-dense foods (chips, fries, candy, sugar-sweetened beverages) was associated with increased intake of sugar-sweetened beverages in children. No significant association existed for parent modeling of dietary intake and child overall Healthy Eating Index-2010 score.
2019	Barriers and facilitators to healthy eating among low-income Latino adolescents	Beck AL; Iturralde EM; Haya-Fisher J; Kim S; Keeton V; Fernandez A	Explore barriers and facilitators to healthy eating among low-income Latino adolescents	Observational, qualitative, cross-sectional	(n = 30) Low-income adolescents (13–17 y)	Adolescents portrayed basic nutritional knowledge however had significant misconceptions about healthy eating. Adolescents reported parents showed support with healthy eating through purchasing healthy foods, providing home-cooked meals, and role modeling but also that family meals were calorie dense and had low nutrient value. Peers were mainly a negative influence in eating habits. Half reported disliking school lunch, and many reported having easy access to unhealthy food near school.
2020	Barriers to preparing and cooking vegetables are associated with decreased home availability of vegetables in low-income households	Landry MJ; Burgermaster M; van den Berg AE; Asigbee FM; Vandyousefi S; Ghaddar R; Jeans M R; Yau A; Davis JN	Examine the association of barriers to buying and preparing/cooking vegetables and how this relationship differs according to food-security status	Observational, quantitative, cross-sectional	(n = 1942) Parent-child dyads (third–fifth grade)	Significant association found between food-security status & availability of vegetables in the home; food-insecure households had a 15% lower availability of vegetables in the home than food-secure households. Food-insecure households were reported as more likely to report barriers to buying/cooking/preparing vegetables. Reported barriers included cost, practical food knowledge, and skills.
2021	Caregiver feeding practices as predictors for child dietary intake in low-income, Appalachian communities	McIver MB; Colby S; Hansen-Petrik M; Anderson Steeves ET	Describe the use of caregiver modeling, dietary intake, and home food availability, and determine their association with fruit, vegetable, and high-sugar/high-fat snack food intake in children	Observational, quantitative, cross-sectional	(n = 174) caregiver-child dyads (2–10 y)	Greater use of caregiver modeling positively predicted child vegetable intake and negatively predicted child intake of high-sugar/high-fat snacks. Higher caregiver dietary intake of fruit and vegetables was a significant predictor of child fruit and vegetable intake. Higher home availability of healthier foods was associated with child fruit and vegetable consumption and home availability of less health foods predicted child’s intake of high-sugar/high-fat snacks.
2020	Describing independent eating occasions among low-income adolescents	Banna J; Richards R; Jones B; Anderson AK; Reicks M; Cluskey M; Gunther C; Hongu NK; Lora K; Misner S; Monroe-Lord L; Topham G; Wong SS; Lim E	Describe independent eating occasions among low-income early adolescents including the environmental context, foods selected, reasons for choosing foods, and parental rules about foods consumed	Observational, qualitative, cross-sectional	(n = 46) adolescents (10–13 y)	About 58% of eating occasions were classified as independent eating occasions with about 65% as snacks. The top 4 most frequently consumed foods at independent eating occasions were sweets, total fruit, dairy, and whole fruit. Reasons for choosing foods included preference, it was the only option, convenience, availability, and food was given by someone else. Health was reported as the least common reason for choosing foods. Parental rules for independent eating occasions were focused on avoiding certain foods and not eating too much.
2014	Determinants of fruit and vegetable intake in low-income children and adolescents	Di Noia, J; Byrd-Bredbenner C	Identify determinants of fruit and vegetable intake in low-income children and adolescents and to identify which determinants are associated with intake	Systematic review	Articles including low-income children and adolescents (10–19 y)	Three determinants of fruit and vegetable were consistently related to intake were race/ethnicity, fruit and vegetable preference, and maternal fruit and vegetable intake.
2019	Diet and physical activity changes among low-income families: perspectives of mothers and their children	Zhen-Duan J; Engebretsen B; Laroche HH	Explore how mothers and their children influence each other’s diet and physical activity	Observational, qualitative, cross-sectional	(n = 18) low-income women with diabetes and their children (10–17 y)	Two approaches to behavior change were identified from the study, collaborative and noncollaborative approaches. A collaborative approach involved accepting change, encouraging each other, abstaining from buying and eating certain foods, leading by example, mutual expectations, and compromise. Noncollaborative approaches were individualistic with poor communication. Barriers identified included resource constraints and lack of information regarding healthy diets and recipes.
2017	What's being served for dinner? an exploratory investigation of the associations between the healthfulness of family meals and child dietary intake	Trofholz AC; Tate AD; Draxten ML; Rowley SS; Schulte AK; Neumark-Sztainer D; MacLehose RF; Berge JM	Investigate the specific foods available at family meals, the overall healthfulness of the meals, and their association with children’s dietary intake	Observational, quantitative, cross-sectional	(n = 120) Families with children (6–12 y)	Foods from protein and high sodium components were included in most meals and over half had foods from dairy and vegetable components, whereas few had fruit, juice or dark green vegetable components, and almost half had added sugar components. Significant association existed between majority of components served at family dinner meals and child overall dietary intake.
2018	Understanding the process of prioritizing fruit and vegetable purchases in families with low incomes: “A peach may not fill you up as much as hamburger”	Askelson NM; Meier C; Baquero B; Friberg J; Montgomery D; Hradek C	Understand the strategies and priorities of families with low-income related to purchasing fruit and vegetables	Observational, qualitative, cross-sectional	(n = 127) Parents of children in third grade	Parents reported 3 common themes considered when purchasing fruit and vegetables: Shopping strategies were a common and involved making lists, basing meals on sale items, coupon use, limiting shopping trips, buying cheap and in season foods and basing shops around pay checks Prioritizing food purchases based on budget involved sale buying, foods to bulk out meals, shelf-life, likely to be eaten, lack of preparation, cost, and health. Paying for fruit and vegetables a theme. High cost was the most commonly mentioned barrier to fruit and vegetable purchasing. Participants indicated that fruit and vegetable are not a high priority when food budgets are tight as of perceived low satiety level but “junk foods” were perceived as cheaper.
2017	“Stretching” food and being creative: caregiver responses to child food insecurity	Burke MP; Martini LH; Blake CE; Younginer NA; Draper CL; Bell BA; Liese AD; Jones SJ	Examine the strategies and behaviors caregivers use to manage household food supply when children experience food insecurity	Observational, qualitative, cross-sectional	(n = 746) Caregivers of children (<18 y)	Behaviors caregivers reported included making changes in foods purchased or obtained for household meals, which involves prioritizing some foods (hot dogs, chicken, rice) and using foods that can be used to bulk out meals (stew, pasta, soups). Monetary and shopping strategies were also used and involved buying food according to price and shopping at budget stores. Other strategies used were making changes in household meal pattern (eg, smaller portions, cut adult portions or adult not eating, and adapting home preparations like using leftovers and freezing meals). Behaviors to decrease/increase specific foods in children’s diet included reducing protein foods (meat, chicken, beef), followed by reducing veg, grains & starches (bread and rice) and increase grains & starches (bread, rice), protein foods (hot dogs, beans), mixed dishes.
2016	A qualitative investigation of parents’ perspectives about feeding practices with siblings among racially/ethnically and socioeconomically diverse households	Berge JM; Trofholz A; Schulte A; Conger K; Neumark-Sztainer D	To describe parent feeding practices with siblings	Observational, qualitative, cross-sectional	(n = 88) Parents with at least 2 children (6–12 y)	Most parents reported that decisions on how to feed siblings impacted the foods prepared for meals. Sibling food preferences, planning meals, and in-the-moment decisions were the most common influencers to food choices. Parents managed picky eating by making one meal or providing food option flexibility. Parents report engaging in different feeding practices based on child weight status. Food restriction was reported to be used if the child was overweight, and pressure-to-eat feeding practices were used when one child was a healthy weight.
2018	A qualitative investigation of how mothers from low-income households perceive their role during family meals	Trofholz AC; Schulte AK; Berge JM	Investigate mother’s role during family mealtimes	Observational, qualitative, cross- sectional	(n = 83) Mothers of children (6–12 y)	Mothers described their roles in family meals as helping children make healthy choices at family meals, making the meal happen, monitoring children’s food intake, managing behaviors at the family meals, making the family meal atmosphere enjoyable, and facilitating conversation.
2020	Qualitative evaluation of drivers of eating decisions among SNAP participants in Mississippi	Gray VB; Hardman AM; Byrd SH	Explore food-related decision patterns among SNAP recipients with regard to barriers to healthy eating, perceptions of healthy eating, and healthy eating strategies	Observational, qualitative, cross-sectional	(n = 126) Female caregivers of children (<13 y)	Major drivers of food selection and preparation were cost, convenience, eating habits, family food preferences. Health was a driver when disease was established. Strategies used by participants included couponing, using sale ads, buying in bulk, freezing, shopping at dollar stores, price matching. Participants reported they used food parenting practices in the home. These practices included encouraging healthy eating and changing habits in relation to purchasing food.
2020	Putting knowledge into practice: low-income women talk about food choice decisions	Palmer SM; Knoblauch ST; Winham DM; Hiller MB; Shelley MC	Explore low-income women’s perceived influences of their food choices	Observational, quantitative and qualitative, cross-sectional	(n = 36) Low-income women (19–50 y)	Main barriers to healthy eating were convenience/preparation time, cost, family food preferences, and limits to food assistance programs. Facilitators to healthy eating included self-efficacy for nutrition change and nutritional and health knowledge. Sometimes, type of food and amount were modified because of not having enough money for food.
2021	Perceived produce availability and child fruit and vegetable intake: the Healthy Communities Study	Moffat LF; Ritchie LD; Gosliner W; Plank KR; Au LE	Determine the association of parent’s perception of the home food environment and child fruit and vegetable intake and BMI and differences by food-security and income status	Observational, quantitative, cross-sectional	(n = 5138) Children and their parents (4–15 y)	Parent perceptions of produce access was linked to household fruit and vegetable availability. Household fruit and vegetable availability was also linked with child fruit and vegetable intake. A higher fruit and vegetable intake among children was related to lower BMI z-score. Weaker relationships were present among children living in food-insecure or low-income households.
2019	Parents as role models: associations between parent and young children’s weight, dietary intake, and physical activity in a minority sample	Coto J; Pulgaron ER; Graziano PA; Bagner DM; Villa M; Malik JA; Delamater AM	Examine the relationship between child and parent BMI, fruit and vegetable consumption, and physical activity levels	Observational, quantitative, cross-sectional	(n = 86) Parent- children dyad (5–7 y)	Most parents were not healthy role models, and most parents and children did not meet guidelines for healthy weight, fruit and vegetable intake, or physical activity. A significant association was found between the healthy parent role model index score and child-reported fruit and vegetable intake, indicating that parents who were healthier role models had children with higher fruit and vegetable intake.
2021	Family function and eating behaviors among Hispanic/Latino youth: results from the Hispanic Community Children's Health Study/Study of Latino Youth (SOL Youth)	Colón-Ramos U; Monge-Rojas R; Smith-Castro V; Wang J; Cheng YI; Perreira KM; Van Horn L; Sotres-Alvarez D; Isasi CR; Gallo LC	Investigate family function, home environment, and eating behaviors among youth	Observational, quantitative, cross-sectional	(n = 1466) Children (8–16 y)	Family support was associated with increased youth fruit and vegetable consumption. Higher youth acculturative stress and increasing youth age was associated with reduced family function and closeness. Household food security was indirectly linked with higher fruit and vegetable intake through family closeness and support. Being older was linked to higher intake of empty calories through family closeness. Authoritarian parenting style was associated with reduced youth fruit and vegetable consumption.
2019	Disrupted relationships, chaos, and altered family meals in food-insecure households: experiences of caregivers and children	Rosemond TN; Blake CE; Shapiro CJ; Burke MP; Bernal J; Adams EJ; Frongillo EA	Investigate the relationships between food insecurity, household chaos, and family meals in the circumstances of food-insecure household	Observational, qualitative, cross-sectional	(n = 20) Caregiver-child dyads (9–15 y)	In food-insecure households, meals varied in frequency, location, and quality, especially when there was less food. Household chaos like conflicts with work and afterschool schedules, little food, child visits, and coping with poverty influenced the frequency and location of meals and strained family interactions during mealtimes. During food shortages, parents reported planning meals to allocate scarce food and using convenience foods.
2016	Eating breakfast together as a family: mealtime experiences and associations with dietary intake among adolescents in rural Minnesota, USA	Larson N; Wang Q; Berge JM; Shanafelt A; Nanney MS	Investigate the prevalence and experience of having family meals at breakfast and to examine the association between meal frequency and adolescent diet quality	Observational, quantitative, cross-sectional	(n = 827) Adolescents (Ninth–Tenth grade)	Family dinner frequency was directly associated with family breakfast frequency. Family breakfast frequency was associated with adolescent involvement in the preparation of breakfast meals and with positive attitudes about mealtime. No association found between family breakfast frequency and total diet quality.
2015	Eating and weight-related parenting of adolescents in the context of food insecurity	Bauer KW; MacLehose R; Loth KA; Fisher JO; Larson NI; Neumark-Sztainer D	Examine differences in the eating and weight-related parenting practices that mothers use with adolescent children in both food-insecure and food-secure households	Observational, quantitative, cross-sectional	(n =2,087) Mother- adolescent dyads (12–17 y)	Mothers from households experiencing low or very low food security were more likely to report that they engage in parental control of their child’s diet, including encouraging children to eat, frequently commenting on child’s weight, restricting eating, and pressuring children to eat compared with mothers with food security.
2019	Food parenting practices in rural poverty context	Sano Y; Routh B; Lanigan J	Understand the influences on food parenting behaviors from a parent’s perspective 1) how do mothers describe shaping their children's food experiences? 2) how do mothers negotiate food parenting practices in the context of rural poverty?	Observational, qualitative and quantitative, cross-sectional	(n = 55) Women with a child (<13 y)	Results report the use of coercive control strategies (high power), and balanced, bidirectional control practices (low power). These coercive control strategies included parents deciding what and how much food and using food rewards to obtain control over their food and behavior. Structure strategies like choosing available foods, offering limited choice, and modeling desired behaviors were used. Bidirectional control through autonomy support was a method of parenting that involved children in food decisions. Food selection and food parenting were informed by their knowledge and intention to promote their child’s healthy eating and their assessment of the child’s weight.
2019	Food parenting practices that influence early adolescents’ food choices during independent eating occasions	Gunther C; Reicks M; Banna J; Suzuki A; Topham G; Richards R; Jones B; Lora K; Anderson AK; da Silva V; Penicka C; Hopkins LC; Cluskey M; Hongu N; Monroe-Lord L; Wong SS	Investigate both parent and child perspectives on parenting practices that influence food choices during independent eating practices	Observational, qualitative, cross-sectional	(n = 44) Parent-child dyads (10–13 y)	Common ways to influence independent eating occasions included setting rules and expectations and managing food availability and accessibility. Other ways to influence eating practices included teaching, pressure to eat, monitoring, and modeling. Children reported that parents had rules about what they could eat and used certain strategies to monitor eating.
2015	Characteristics of youth food preparation in low-income, African-American Homes: associations with Healthy Eating Index Scores	Sattler M; Hopkins L; Anderson Steeves E; Cristello A; McCloskey M; Gittelsohn J; Hurley K	Gain insight into food preparation among the youth, including how often they are preparing their own food, what techniques are being used, what associations are there between age, gender, and food preparation, and what is the association between youth food prep and dietary quality	Observational, quantitative, cross-sectional	(n= 289) Child-adult dyads (9–14 y)	Main food prepared in the home required basic skill, few ingredients, little equipment, easily found ingredients/foods in urban food environment. The most common method of preparation was raw preparation, microwaving, and frying. No association was found between frequency of youth food preparation and total HEI score, HEI sodium, empty calories, or dairy scores. Older age and male sex were associated with lower HEI score.
2017	Determinants of sugar-sweetened beverage consumption among low-income children: are there differences by race/ethnicity, age, and sex?	Tasevska N; DeLia D; Lorts C; Yedidia M; Ohri-Vachaspati P	Identify child and parent lifestyle and household demographic factors that predict high sugar-sweetened beverage intake frequency in children from low-income, ethnically diverse communities to inform public health interventions	Observational, quantitative, cross-sectional	(n = 1,403) Parents of children (3–18 y)	Factors linked to lower sugar-sweetened beverage consumption included living in a non-English speaking household, low parental consumption, having a parent with college education or higher, and having moderate consumption of breakfast of 6–7 d/wk compared with 0–2 d/wk. Older children compared to younger had higher intake as did non-Hispanic black compared with white. Six to 11 year olds who were moderate/high consumers of breakfast were 15%–20% points less likely to be in the highest sugar-sweetened beverage category, and 3%–6% more likely to never consume.
2021	Examining factors related to the food insecurity-obesity paradox in low-income mothers and fathers	Taylor EA; Foster JS; Mobley AR	Examine the factors that may be related to the gender disparity in the food insecurity-obesity paradox	Observational, qualitative and quantitative, cross-sectional	(n = 25) Mother and father pairs with their child (2.5–10 y)	Results report that parents sacrificing their own diet quality to feed children was a common strategy used in the households. Mothers were significantly more likely to restrict their own food to ensure their children had enough to eat compared to fathers. Strategies parents used to ensure children obtaining enough food included letting children eat their meal first and splitting the remainder, prioritizing where money is spent, or eating something else if there is not enough of the meal.
2020	Factors related to poor diet quality in food-insecure populations	Ranjit N; Macias S; Hoelscher D	To examine food procurement related behaviors and psychosocial attitudes in food-insecure populations	Observational, quantitative, cross-sectional	(n = 1171) Adults (≥18 y)	The food-insecure compared with food-secure group had less healthful diets with less frequency and serving size of fruits and vegetables, were more likely to use cost saving practices, such as comparing prices, and less likely to cook/eat a home-cooked meal or read food labels compared with food secure. There was little difference in anticipatory behaviors between households such as meal planning, making a list. Self-efficacy for healthy eating and planning meals with vegetables were lower among the food-insecure vs food-secure group.
2016	Family chaos and lack of mealtime planning is associated with food insecurity in low-income households	Fiese BH; Gundersen C; Koester B; Jones B	Examine the role of family chaos and mealtime planning in food-insecure and food-secure households	Observational, quantitative, cross-sectionally analyzed	(n = 221) Parents of elementary school children	Family chaos is statistically significant and positively associated with food insecurity. Food-insecure households reported less meal planning than food-secure households.
2019	Food and financial coping strategies during the monthly Supplemental Nutrition Assistance Program cycle	Kinsey EW; Oberle M; Dupuis R; Cannuscio CC; Hillier A	Explore the nature and timing of coping strategies for managing the SNAP cycle and implications coping mechanisms have for health and financial stability	Observational, qualitative and quantitative, prospective cohort	(n = 12) Mothers receiving the SNAP	Strategies reported to manage the SNAP benefits included: • Adjustments to shopping and eating patterns like adjusting purchasing at each shopping trip, cooking meals at home, menu planning, making shopping lists, buying sale items, couponing, using shopping outlets based on sales/promotions. • Eating less/skipping meals when food is running out to make sure children have enough. • Price and quantity are major determinants. • Mental accounting, constant vigilance, and resilience for food budgeting. • Reliance on social network for emotional and informational support or support through resources.
2016	Food insecurity, overweight and obesity among low-income African-American families in Baltimore City: associations with food-related perceptions	Vedovato GM; Surkan PJ; Jones-Smith J; Anderson Steeves E; Han E; Trude AC; Kharmats AY; Gittelsohn J;	Investigate links between food insecurity, excess body weight, psychosocial factors, and food behaviors	Observational, quantitative, cross-sectional	(n = 298) Caregiver-child dyads (10–14 y)	Patterns of food sources used, food acquisition, preparation, knowledge, self-efficacy, and intentions were not different by food security. Food-secure households had higher level of agreement with healthy food as being affordable when compared with both food-insecure groups and food-insecure groups who experienced hunger. The food-insecure group reported healthy food as being less accessible and less convenient to buy and prepare.
2014	Food preparation supplies predict children’s family meal and home-prepared dinner consumption in low-income households	Appelhans BM; Waring ME; Schneider KL; Pagoto SL	Investigate the association of food preparation supplies availability in the home with family meal frequency and child consumption of home-prepared meals	Observational, quantitative, cross-sectional	(n = 103) Caregivers of child (6–13 y)	Higher food preparation supplies associated with more frequent family meals and child intake of home-prepared meals. More frequent family meals and intake of home-prepared meals was linked to more healthful dietary intake such as higher intake of fruit and vegetables, and reduced sugar-sweetened beverage. Financial strain was associated with reduced family meal frequency.
2018	Friends and family: how African-American adolescents’ perceptions of dietary beliefs and behaviors of others relate to diet quality	Wrobleski MM; Parker EA; Hager E; Hurley KM; Oberlander S; Merry BC; Black MM	Determine if perceived parental dietary beliefs, caregiver-reported parental monitoring of the adolescent diet, and perceived peer eating behaviors are related to adolescent diet quality	Observational, quantitative, cross-sectional	(n = 216) Caregiver-adolescent dyads (11–16 y)	Dietary quality scores among adolescents are positively associated with adolescents’ perceptions of parents’ beliefs regarding nutrition, healthy dietary choices among peers, and caregivers report of parental monitoring of adolescent dietary behavior. Adolescents’ dietary behaviors reflect their perceptions of the social environment.
2020	What a city eats: examining the dietary preferences of families living in communities at high risk for food insecurity	Cummer E; Loyola Amador C; Montez K; Skelton JA; Ramirez B; Best S; Zimmer R; Palakshappa D	Evaluate the dietary patterns, food preferences, and meal preparation methods of families at high risk of food insecurity	Observational, qualitative, cross-sectional	(n = 63) Adults	Sample population showed food preferences with a high intake of calorie dense foods and low consumption of vegetables. Expressed little interest in learning new recipes or cooking methods to assist with healthy eating.
2015	Rural Latino caregivers’ beliefs and behaviors around their children’s salt consumption	Hoeft KS; Guerra C; Gonzalez-Vargas MJ; Barker JC	Examine the knowledge, beliefs, and behaviors of rural Latino caregivers regarding their children’s salt consumption and related health implications	Observational, qualitative, cross-sectional	(n = 61) Caregivers of children (elementary school age or less)	Caregivers recognized sources of sodium and reported they used strategies to reduce their child’s sodium intake; for example, limiting salt used in food preparation, limiting frequency of salty food consumption. However, caregivers overlooked other significant sources of salt like bread, cheese, soup, and sports drinks.
2018	Psychosocial determinants of food acquisition and preparation in low-income, urban African-American households	Henry JL; Trude ACB; Surkan PJ; Anderson Steeves E; Hopkins LC; Gittelsohn J	Identify psychosocial factors influencing food purchasing and food preparation behaviors of adult caregivers in African-American low-income urban communities	Observational, quantitative, cross-sectional	(n = 465) Caregivers of children (10–14 y)	Higher food-related behavioral intention scores were marginally associated with healthier food acquisition, healthier food preparation methods, and lower frequency of purchasing at prepared food sources. Greater food-related self-efficacy was associated with healthy food preparation methods and negatively associated with purchasing at prepared food sources. Higher nutrition knowledge was only associated with lower frequency of purchasing at prepared food sources.
2019	Parenting styles are associated with overall child dietary quality within low-income and food-insecure households	Burke MP; Jones SJ; Frongillo EA; Blake CE; Fram MS	Examine the association between parenting styles and child dietary quality in low-income and food-insecure households	Observational, quantitative, cross-sectional	(n = 171) Parent-child dyads (9–15 y)	Authoritative and authoritarian parenting styles had a significant association such that as parents reported more authoritative parenting attitudes and behaviors, their children were predicted to have higher dietary quality if they also reported average or greater authoritarian attitudes and behaviors. Permissive parenting attitudes and behaviors were negatively associated with child dietary quality.
2016	Low-income mothers’ feeding goals predict observed home mealtime and child feeding practices	Pesch MH; Miller AL; Appugliese DP; Kaciroti N; Rosenblum KL; Lumeng JC	Examine the association of mothers feeding goals with observed home mealtime and feeding practices	Observational, quantitative, longitudinal	(n = 265) Female caregiver-child dyads (mean age of child: 70.8 mo)	Goal of restricting junk food associated with child always eating at the table, but not with mother restricting junk food. Goal of promoting fruit and vegetable associated with mother promoting vegetables. Goals of promoting autonomy and preventing obesity not associated with home mealtime or feeding practices. Parental feeding goals may not turn into feeding practices.
2022	Kitchen adequacy and child diet quality in a racially/ethnically diverse sample	Fertig AR; Trofholz AC; Loth K; Tate AD; Miner M; Neumark-Sztainer D; Westfall EC; Westby A; Berge JM	Investigate the kitchen adequacy of households with young children from low socioeconomic backgrounds with child dietary quality	Observational, quantitative, cross-sectional	(n =149) Families with children (5–7 y)	The majority of families had adequate kitchen facilities and supplies in their homes. A kitchen table was associated with higher dietary quality among children. Can openers and measuring spoons present in the household were linked to higher sodium and added sugars, respectively. Results suggest that kitchen adequacy is not a major barrier to overall healthy eating.
2020	Is healthy eating too expensive? How low-income parents evaluate the cost of food	Daniel C	Investigate how low-income consumers evaluate the cost of food	Observational, qualitative, cross-sectional	(n = 34) Caregivers of children (primarily 4–8 y)	Findings report that participants judged the cost of food in 2 ways • Absolute judgment regarding if food would meet family needs with few resources • Relative judgment or evaluation of price in comparison to other foods that make an item affordable or expensive by contrast
2019	“I try, I do:” Child feeding practices of motivated, low-income parents reflect trade-offs between psychosocial- and nutrition-oriented goals	Schuster RC; Szpak M; Klein E; Sklar K; Dickin KL	Understand parents’ goals for feeding their children, underlying motivations for these goals, the strategies they employed to work toward them, and the contextual environment that challenged or facilitated achievement of these goals	Observational, qualitative, cross-sectional	(n = 21) Caregiver of child (3–11 y)	Nutrition and health-oriented goals were reported by parents, which included encouraging child to eat nutritious diet, fostering healthful relationship with food, economizing food costs, reducing parents’ own meal size/frequency, and avoiding inadequate nutrient intake. Psychosocial-oriented goals included having family meals to enhance family relationships and helping child feel secure. Sometimes psychosocial goals were in conflict with nutrition-oriented goals, like giving into child food preferences to avoid conflict or preserve self-esteem.
2017	How parents describe picky eating and its impact on family meals: a qualitative analysis	Trofholz AC; Schulte AK; Berge JM	Examine parents’ experiences and perspectives regarding picky eating in order to understand its impact on families, including during family meals	Observational, qualitative, cross-sectional	(n = 88) Caregivers of children (2–18 y)	Picky eating was reported to be disruptive to family meals, creating meal-related stress. Picky eating can lead to altering meal preparation or making separate meals. Strategies parents use in response to picky eating include making child try the food, making separate meal, using take it or leave it approach, or child makes separate meal.
2016	Household, psychosocial, and individual-level factors associated with fruit, vegetable, and fiber intake among low-income urban African-American youth	Trude ABC; Kharmats AY; Hurley KM; Anderson Steeves E; Talegawkar SA; Gittelsohn J	Identify the characteristic, psychosocial, and household factors influencing fruit and vegetable consumption in low-income African-American youth	Observational, quantitative, cross-sectional	(n = 285) Caregiver-youth dyads (10–14 y)	Fruit, vegetable, and fiber intake were positively related to youth intentions and self-efficacy for eating healthy. Youth who received free/low-cost breakfast were more than 2 times as likely to have high fiber intakes as those who did not receive free breakfast. Youth purchasing food at supermarkets was associated with an increase in vegetable serving and fiber intakes. Youth with parents who purchase food at fast-food restaurants showed a 7% decrease in odds for vegetable intake. Self-efficacy, health outcome expectations, intention, and knowledge are important psychosocial factors that may influence eating behavior.
2016	Home food environment factors associated with the presence of fruit and vegetables at dinner: a direct observational study	Trofholz AC; Tate AD; Draxten ML; Neumark-Sztainer D; Berge JM	Investigate what home food environment characteristics are associated with the presence of fruit and vegetables at family dinners	Observational, quantitative, cross-sectional	(n = 120) Families with children (6–12 y)	Home availability and accessibility of fruits and vegetables were predictive of fruit and vegetable intake in children and adolescents. Meal planning was linked with the presence of fruit at dinners. Higher parent intake of vegetables was associated with vegetables at dinner.
2013	Eat this, not that! Parental demographic correlates of food-related parenting practices	Loth KA; MacLehose RF; Fulkerson JA; Crow S; Neumark-Sztainer D	Investigate how food-related parenting practices, specifically how restriction and pressure to eat among parents of adolescents differ across sociodemographic characteristics	Observational, quantitative, cross-sectional	(n = 3709) Parents/guardians of adolescents	Parental control over their adolescent regarding how much food to eat, as well as what types of foods the adolescent should avoid was common, especially among parents of race/ethnic minority subgroups, those with less than a high school education, and those with low household income.
2022	Diet quality and contextual factors influencing food choice among adolescents with food security and food insecurity in Baltimore City	Harper K; Caulfield LE; Lu SV; Mmari K; Gross SM	Evaluate differences in overall diet quality and food-related contextual factors between adolescents with food security and insecurity	Observational, quantitative and qualitative, cross-sectional	(n = 61) Adolescents (14–19 y)	No significant differences in overall diet quality or components between food-security groups except for seafood and plant proteins that was higher for food-insecure adolescents, adolescents were influenced by having food available at home and eating family meals.

BMI, body mass index; HEI, Healthy Eating Index; SNAP, Supplemental Nutrition Assistance Program

**TABLE 2:** Themes identified among 44 studies addressing household factors of dietary quality and food security in US households of school-aged children from PubMed, years 2012 to December 2022.

Study Title	Parental Behaviors	Adolescent /Child Behaviors	Food Procurement Behaviors	Food Preparation Behaviors	Household Environment Factors
Associations between parent and child physical activity and eating behaviors in a diverse sample: an ecological momentary assessment study	X				
Barriers and facilitators to healthy eating among low-income Latino adolescents	X	X			
Barriers to preparing and cooking vegetables are associated with decreased home availability of vegetables in low-income households			X	X	
Caregiver feeding practices as predictors for child dietary intake in low-income, Appalachian communities	X			X	
Characteristics of youth food preparation in low-income, African-American homes: associations with healthy eating index scores				X	
Describing independent eating occasions among low-income adolescents	X	X			
Determinants of fruit and vegetable intake in low-income children and adolescents	X	X			
Determinants of sugar-sweetened beverage consumption among low-income children: **are**there differences by race/ethnicity, age, and sex?	X	X			
Diet and physical activity changes among low-income families: perspectives of mothers and their children	X				
Disrupted relationships, chaos, and altered family meals in food-insecure households: experiences of caregivers and children				X	X
Eating breakfast together as a family: mealtime experiences and associations with dietary intake among adolescents in rural Minnesota, USA	X	X		X	X
Eat this, not that! Parental demographic correlates of food-related parenting practices	X				
Eating- and weight-related parenting of adolescents in the context of food insecurity	X				
Examining factors related to the food insecurity-obesity paradox in low-income mothers and fathers	X				
Family chaos and lack of mealtime planning is associated with food insecurity in low-income households				X	X
Family function and eating behaviors among Hispanic/Latino youth: results from the Hispanic Community Children's Health Study/Study of Latino Youth (SOL Youth)	X				X
Food and financial coping strategies during the monthly Supplemental Nutrition Assistance Program cycle	X		X		
Food insecurity, overweight and obesity among low-income African-American families in Baltimore City: associations with food-related perceptions	X			X	
Food parenting practices that influence early adolescents’ food choices during independent eating occasions	X				
Food parenting practices in rural poverty context	X				
Food preparation supplies predict children's family meal and home-prepared dinner consumption in low-income households				X	X
Friends and family: How African-American adolescents’ perceptions of dietary beliefs and behaviors of others relate to diet quality	X	X			
Home food environment factors associated with the presence of fruit and vegetables at dinner: **a** direct observational study			X	X	
Household, psychosocial, and individual-level factors associated with fruit, vegetable, and fiber intake among low-income urban African-American youth		X			
How parents describe picky eating and its impact on family meals: **a** qualitative analysis		X			
**“**I try, I do**”:** Child feeding practices of motivated, low-income parents reflect trade-offs between psychosocial- and nutrition-oriented goals	X				X
Factors related to poor diet quality in food-insecure populations	X		X	X	
Is healthy eating too expensive?: How low-income parents evaluate the cost of food			X		
Kitchen adequacy and child diet quality in a racially/ethnically diverse sample				X	
Low-income mothers’ feeding goals predict observed home mealtime and child feeding practices	X				
Parenting styles are associated with overall child dietary quality within low-income and food-insecure households	X				
Parents as role models: associations between parent and young children's weight, dietary intake, and physical activity in a minority sample	X				
Perceived produce availability and child fruit and vegetable intake: the Healthy Communities Study				X	
Psychosocial determinants of food acquisition and preparation in low-income, urban African-American households	X		X	X	
Putting knowledge into practice: low-income women talk about food choice decisions	X		X	X	
Qualitative evaluation of drivers of eating decisions among SNAP participants in Mississippi	X		X		
A qualitative investigation of how mothers from low-income households perceive their role during family meals	X				
A qualitative investigation of parents’ perspectives about feeding practices with siblings among racially/ethnically and socioeconomically diverse households	X	X			
Rural Latino caregivers’ beliefs and behaviors around their children's salt consumption	X				
Stretching food and being creative: caregiver responses to child food insecurity	X		X	X	
Understanding the process of prioritizing fruit and vegetable purchases in families with low incomes: **“**A peach may not fill you up as much as hamburger**”**			X		
What a city eats: examining the dietary preferences of families living in communities at high risk for food insecurity	X				
What’s being served for dinner? An exploratory investigation of the associations between the healthfulness of family meals and child dietary intake				X	
Diet quality and contextual factors influencing food choice among adolescents with food security and food insecurity in Baltimore City	X	X		X

## Results

All of the 44 studies were observational, and the majority were published in the last 5 years (*n* = 27, 61%); used a cross-sectional (*n* = 41, 93%) or prospective/longitudinal (*n* = 2, 5%) study design; and used quantitative (*n* = 23, 52%), qualitative (*n* = 15, 35%), or a mixed methods (quantitative and qualitative) approach (*n* = 5, 11%). One of the studies was a systematic review (*n* = 1, 2%). Data was collected from parents/caregivers (*n* = 19, 43%), parent/caregiver and child dyads (*n* = 15, 34%), adolescents (*n* = 6, 14%), and families (*n* = 4, 9%). Several studies addressed more than 1 of the 5 themes: *1*) parental behaviors, *2*) adolescent/child behaviors, *3*) food procurement behaviors, *4*) food preparation behaviors, and *5*) household environment. For example, 31 studies (70%) included parental behaviors, 10 studies (23%) included adolescent/child behaviors, 10 studies (23%) included food procurement behaviors, 17 studies (39%) included food preparation behaviors, and 6 studies (14%) included household environment factors.

Evidence of the theme, parental behaviors, related to dietary quality and food insecurity in low-income US households with children included behaviors of parental modeling, feeding practices, attitudes and support, parental self-efficacy, knowledge, and parenting styles. Each of these behaviors and the evidence of their relationship to dietary quality and food security are described below. Parental modeling, or a parent’s effort to demonstrate healthy food choices and healthy eating behaviors with the objective that the child will exhibit similar behaviors, was included in 11 of the studies [[30], [31], [32], [33], [34], [35], [36], [37], [38], [39], [40]]. Five studies showed parental modeling was a support to encourage overall healthy eating behaviors among children [[36], [37], [38], [39], [40]], whereas 5 studies showed a more specific association to increased fruit and vegetable intake [[30], [31], [32], [33], [34]], and one showed healthy eating modeling was associated with reduced child consumption of high-sugar/high-fat snacks [34]. Alternatively, modeling intake of energy-dense food was associated with increased sugar-sweetened beverage intake among children in 4 studies [31,[33], [34], [35]]. Parental feeding practices such as encouragement, pressure to eat, food restriction, and controlling food intake, based on child characteristics like weight, age, and developmental stage, were other parental behaviors contributing to dietary quality and food security in the household [[38], [39], [40], [41], [42], [43], [44], [45], [46]]. Food restriction was a reported practice when the child was overweight, and pressure to eat was reported when the child was at a healthy weight [38,39,41]. Low-income households and those with low or very low food security were more likely to engage in food restriction and pressure-to-eat practices compared with higher-income and food-secure households [42,43]. Parental eating attitudes, teaching, and support toward healthful diets also played a role in eating habits among children [39,40,44,45]. Practices related to attitudes, teaching, and support included limiting the availability of sugar-sweetened foods in the home, establishing healthy parental relationships with food, encouraging children to make healthy choices, and family eating practices like eating meals together [39,40,[44], [45], [46], [47], [48]]. Parents from low-income households expressed goals to restrict junk food and encourage a nutritious diet including fruit and vegetable intake [45,49] but also reported that not all parental feeding goals turned into practices [49]. For example, parents reported buying foods that they knew children would eat to avoid wasting food, which could contribute to purchasing foods with lower dietary quality [44]. When food resources were low, parents sometimes adapted the types of food served at meals and the amount [50,51]. In food-insecure situations, parents reported reducing their own portion sizes and skipping meals to ensure food was available for children [45,[50], [51], [52], [53]]. Several studies discussed the influence of parent or caregiver self-efficacy for healthful diets and nutritional and health knowledge of dietary quality and/or food security [51,[54], [55], [56], [57]]. Self-efficacy, which refers to a person’s belief in their ability to engage in healthy eating behaviors, was associated with increased dietary quality and healthy eating behaviors in low-income families and adolescents [51,55). Nutritional knowledge [36,54,56] was also identified as a factor in dietary quality. For example, caregivers had a basic understanding of sodium sources but had difficulty identifying sodium in sources, such as cheese and prepared soups, and little knowledge of the effects of increased salt consumption on childhood health [56]. The relationship of food insecurity to self-efficacy for healthy eating was less clear as 2 studies showed conflicting results [54,58], and food insecurity did not differentiate food knowledge or intentions [58]. Parenting style was the last area of parental behavior showing links to dietary quality in children in 2 studies [59,60]. One showed that authoritarian parenting attitudes and behaviors including high demands, low responsiveness, low emotional warmth, and unwillingness to negotiate were positively associated with the dietary quality of the child [59]. The same study also showed permissive parenting styles with few rules and freedom of child dietary choice were negatively associated with child dietary quality [59]. However, the second study showed conflicting results with an association between authoritative parenting style and reduced fruit and vegetable consumption among adolescents [60]. Yet, both studies showed parenting style as a potential determinant associated with nutritional quality. Therefore, parenting style along with all other parental behaviors reviewed have links to dietary quality and food security that also linked them to the other themes identified in the review and are likely influential in food procurement and preparation (Figure 2).

Adolescent/child behaviors in 10 studies [30,33,35,36,41,[46], [47], [48],^61^,^62^] included investigations of personal food preferences; healthy eating knowledge, attitudes and self-efficacy; picky eating behaviors; and involvement in food preparation. Adolescents reported choosing food based on preference, convenience, and/or the foods available in the home or elsewhere [30,46,48]. Health was considered the least common reason for food choice [46]. Adolescents were able to demonstrate basic nutrition knowledge and recognized healthy and unhealthy foods but still held some misconceptions around healthy foods [36]. Healthy eating intentions, self-efficacy, and purchasing food at supermarkets were linked to adolescent fruit, vegetable, and fiber intake [61] whereas poor eating behaviors like high intake of fast food and low breakfast intake were linked with other poor dietary practices like high sugar-sweetened beverage intake [35]. The dietary quality of adolescents was linked to their perceptions of adult nutrition beliefs and peer dietary choices [33], which were a negative influence on eating habits [36]. Adolescents motivated to adopt healthy dietary practices reported that it was difficult to sustain healthy eating changes [36]. Picky eating behaviors among children were also included in the studies and found to be disruptive on family mealtime and the home environment, causing meal-related stress and increased time parents spend in meal preparation to adjust or make additional meals [[41], [63]]. However, adolescent involvement in the preparation of breakfast and positive attitudes about mealtime were linked to family breakfast frequency [47]. Similar to the parent behaviors identified in the review, child behaviors overall had relationships with the other themes and are likely influential in food procurement and preparation.

Food procurement behaviors in 10 studies [44,[50], [51], [52],^54^,^55^,^63^,[64], [65], [66]] comprised topics of purchasing behaviors and strategies and barriers to purchasing foods. Priorities that individuals considered when purchasing foods included cost, family preferences, food preparation time, family needs, and shelf-life. Price was a major determinant in purchasing behaviors [52] and more often reported in food-insecure compared with food-secure households [65]. Strategies to help save money included making lists, buying sale items, buying in bulk, comparing prices, shopping in discount stores, limiting trips, using certain foods to “bulk out” meals, and using coupons [44,[50], [51], [52],^54^,^63^,^64^]. Food-insecure compared with food-secure households were significantly more likely to use cost saving practices, such as price comparisons [54]. However, there was no difference between the groups in the use of anticipatory behaviors, including meal planning and making a shopping list [54]. The high cost of fruit and vegetables was reported as a main barrier to their procurement [63,65], but another study found no relationship between purchasing barriers and the presence of fruit and vegetables at dinner meals [66]. Acquisition of healthy foods was linked to higher caregiver food-related behavioral intentions and self-efficacy and lower frequency of purchasing at prepared food sources, which was also linked with higher caregiver nutrition knowledge [55], exemplifying how parental/caregiver behaviors are related to food procurement. Food procurement is also likely to influence food preparation as individuals purchase foods that they plan to prepare, but food preparation factors may also change and influence food procurement.

Food preparation factors and behaviors like meal planning, having kitchen/cooking supplies and time to perform these behaviors, cooking skills to carry out food preparation, and food availability were addressed in 17 studies [34,47,48,50,51,54,55,58,[65], [66], [67], [68], [69], [70], [71], [72], [73]]. Several studies investigated meal preparation and planning behaviors [47,51,54,55,58,65,66,68,69,72,73]. Adult/caregiver food-related self-efficacy was linked with healthy food preparation methods [55]. Food-insecure households were more likely to report barriers to cooking and preparing vegetables or healthy food [58,65] and less likely to plan [73] and prepare a home-cooked meal compared to food-secure households [54]. Furthermore, low-income households, regardless of food-security status, had a low prevalence of planning behaviors related to buying and preparing food [54]. Lack of sufficient time was identified as a barrier to making home-cooked meals [51]. The accessibility of food preparation supplies [67,70] was associated with the frequency of family meals, and more frequent family meals were linked with more healthful dietary intake, such as greater fruit and vegetable intake and less sugar-sweetened beverage intake [48,67]. Contradictorily, a longitudinal observational study’s results suggested that kitchen adequacy was not a barrier to healthy eating [70]. Youth also prepared food at home [48] using basic skills, a few pieces of cooking equipment, and easily accessible ingredients [68], and their involvement was linked to family breakfast frequency [47].The availability of healthy foods was associated with dietary quality and food security in 6 studies [34,48,65,66,70,71] and specifically increased fruit and vegetable intake [34,48,66,71,72]. Food-security status had additional links with the availability of vegetables in the home [65], barriers of knowledge of how to prepare and cook vegetables [65], and using strategies to manage the household food supply like using leftovers and freezing meals [50] and planning meals to allocate scarce food [69]. Such tactics suggest a bidirectional link from food preparation to food procurement and links to the themes of adult/parent and child behaviors.

Finally, the household environment and specifically family chaos was identified as an influencing factor on dietary quality and food security, owing to its negative impact on family relationships and mealtime-related stress in 6 studies [45,47,60,67,69,73]. Household chaos included work and schedule conflicts, food shortages, coping with poverty, and children visiting multiple homes, which reportedly influenced the frequency and locations of meals [67,69]. Family dinner frequency was directly linked with family breakfast frequency, which was linked with positive attitudes about mealtimes [47]. A qualitative study found that aiming to have family meals to enhance family relationships and helping children feel secure in the home may assist in overcoming mealtime chaos [45]. However, sometimes prioritizing making children feel secure was in conflict with nutrition-oriented goals like acquiescing to child food preferences to maintain harmony or promote self-esteem [60]. Yet, family support was associated with increased youth fruit and vegetable intake, and household food security had an indirect link to higher fruit and vegetable intake through family closeness and support [60]. The household environment, then, and specifically the family chaos of coping with poverty, is likely to influence both parent and child behaviors along with the food procurement and food preparation in the household.

None of the studies explored all 5 of the themes of household factors linked to dietary quality and food security nor their various relationships, yet the connections between these 5 themes are conceptualized by the authors to be numerous and complex. Figure 2 summarizes the potential relationships or directionality of influence of the themes based on the findings within the studies. In some cases, the relationships are likely bidirectional; for example, the household environment may be both influential on behaviors of household members and influenced by those behaviors. In other cases, only one-directional relationships may be present, as in how parental behaviors likely drive food procurement with influence from children and how parental behaviors may drive food preparation similarly with influence from children.

## Discussion

This scoping review identified 5 themes of household factors covered in the existing literature that are linked to dietary quality and food security among low-income households with school-aged children and explored how various relationships exist among these themes. Mapping the relationships of the themes reveals new insights to apply to interventions and programs aimed at improving food security and dietary quality within the outer levels of the social ecological model. The themes identified in this review may be conceptualized within the inner levels of the social ecological model. For example, dietary quality and food security are experienced by the individual. Next, the parent and child behaviors exert direct influence on individuals within the interpersonal relationships level, which includes the family members in a person’s closest circle contributing to their experiences. Food procurement and preparation are specific food-related behaviors by household members that influence those within the household, and the household environment encompasses the family dynamics, chaos or order, habits, and schedules that also exert influence on individual diet quality and food security. Evidence of themes in this review and their potential application to improve diet quality and food security is discussed below.

Parental behaviors, included in the majority of studies, such as parental modeling, support and encouragement, feeding practices, and parenting styles, highlight the significant influence parents have on the food their child is consuming, including the amount and types of foods [[30], [31], [32], [33], [34], [35], [36], [37], [38], [39], [40], [41], [42], [43], [44], [45], [46], [47], [48], [49], [50], [51], [52], [53], [54], [55], [56], [57], [58], [59], [60]]. Parental modeling of healthy eating appears an important contributor to dietary quality and food insecurity because of the consistency among studies showing a positive impact on their child’s eating behaviors [[30], [31], [32], [33], [34],[36], [37], [38], [39], [40]]. Additionally, parental support and encouragement for healthy eating can improve children’s attitudes toward healthy eating whereas food restriction and pressure to eat can result in a child’s negative attitude toward food and meals [[38], [39], [40], [41], [42], [43], [44], [45], [46]]. Children’s behaviors had less coverage in studies compared with parental behaviors, and therefore there less evidence; however, children were shown as having an influential role in their dietary quality and household food security [30,33,35,36,41,[46], [47], [48],^61^,^62^]. Their behaviors, preferences, self-efficacy, and nutrition knowledge were linked to parental behaviors, food purchasing, and preparation in the household. Self-efficacy for healthy eating behaviors among both parents [51,55] and children [61] was linked with healthful dietary intake. Considering the role parents play in managing food resources, influencing children, and the household environment, and the role that children also play in parental behaviors and decisions on food purchasing and preparation, future interventions to food insecurity and dietary quality of household members should address parent and child behaviors, attitudes, and preferences.

Food procurement [44,[50], [51], [52],^54^,^55^,^63^,[64], [65], [66]] and preparation factors [34,47,48,50,51,54,55,58,[65], [66], [67], [68], [69], [70], [71], [72], [73]] were also themes linked to dietary quality and food security in this review. Findings suggest that low-income and food-insecure households experience more barriers to purchasing and preparing foods, including lack of access to foods and challenges with the price of food, when compared with households of higher-income and food-secure households [52,54,58,65,73]. Overcoming barriers to purchasing and preparing foods may assist in improving the availability of food within the household. Several studies documented the use of strategies in purchasing foods, for example comparing prices, buying sale items, and shopping in budget stores, to assist parents in managing their budgets [44,[50], [51], [52],^54^,^63^,^64^]. Similarly, many studies [34,48,65,66,71,72] also associated availability of healthy foods in the home with increased dietary quality and increased consumption of fruit and vegetables, which is consistent with previous studies and reviews [74,75], whereas the availability of less healthy foods in the home was linked with increased child intake of high-sugar/high-fat snacks [76]. Therefore, applications to improve the availability of healthful foods and decrease access to less healthful foods in the household may enhance the dietary quality of children and food security.

The household environment may also play a role in food security and dietary quality of household members as the chaos or order in the home can impact plans for food purchasing and preparation [45,47,60,67,69,73]. Furthermore, the household environment may impact how much control, self-efficacy, and other behaviors parents and children have toward dietary intake and ensuring access to food. However, fewer studies contributed to the evidence of the relationships of various themes with the household environment despite these expected relationships, representing a gap in the literature and a contribution of this scoping review. The interactions between the household-level factors of household environment, parent and child behaviors, and food procurement and preparation may ultimately work together to impact dietary quality and food security for all within a household. For example, lack of nutritional knowledge, food preparation, and cooking skills with a low self-efficacy for healthy eating among parents and children and a chaotic household can act as barriers to healthy eating and reduce the dietary quality of foods/meals consumed in the home and contribute to feelings of stress with regard to obtaining enough food in the household when resources are also low. Challenges with these skills may contribute to the higher prevalence of food-insecure groups eating away from home and reduced prevalence of food-insecure groups eating home-cooked meals [54]. However, the potentially complex nature of how the various factors influence food insecurity and dietary quality synergistically should be addressed in future studies.

## Implications

The evidence summarized has implications to the organizational, community, and policy levels of the social ecological model. Individuals from low-income households participate in federal US nutrition assistance programs, such as the Supplemental Nutrition Assistance Program (SNAP) [77], which provide participants with financial benefits to assist with food purchasing and operate through organizations, communities, and policy. SNAP resources are external to the household and can play an important role in supporting households to improve food security [21]. SNAP eligibility also allows individuals to participate in nutrition education programs such as SNAP-Education (SNAP-Ed), evidence-based nutrition education program focusing on nutrition, budgeting, and a healthy lifestyle [[78], [79], [80], [81], [82], [83], [84]]. Nutrition education programs target household factors, such as nutrition behaviors, attitudes, and knowledge, and can play a significant role in improving food security [[79], [80], [81], [82],^84^] with limited evidence regarding dietary quality [81,83]. In other studies, nutrition education has been shown to improve participants’ food security over 1 year after receiving the program [79,81,84]. However, less evidence has shown nutrition education improvement on dietary quality despite aims to help align household dietary choices with the recommendations in the DGA [80,82]. The results of this scoping review can be applied to future nutrition education efforts to improve healthful dietary habits and behaviors by informing educational content.

Nutrition education may include behaviorally focused programming through direct nutrition education that promotes dietary strategies to maximize the intake of nutrient-dense foods and beverages while stretching food dollars [78]. This review identified additional topics to be considered for inclusion in future nutrition education programming for households with school-aged children such as addressing parent and child behaviors and attitudes. Involvement of children in adult lessons and recognition of their influence on household food procurement, purchasing, and parent behaviors may promote parents and children to jointly learn about nutrition, improve attitudes toward healthful foods, and promote self-efficacy to support dietary quality. Similarly, educating parents on the importance of modeling healthy eating behaviors in the home and providing their children with encouragement and support may also build healthful habits. Promoting early exposure to a range of nutrient-dense foods, including fruits and vegetables, and having these foods available in the home should be encouraged as it may improve diet quality in future life stages and promote positive attitudes toward eating throughout childhood and adolescence. In addition, future nutrition education lessons should consider educating parents on the implications of feeding practices, such as pressure to eat and food restriction, on their child’s dietary quality.

The food procurement and preparation themes identified in this review are addressed in current nutrition education programs such as by teaching practical strategies to improve cooking skills, meal selection, and planning, and improve attitudes toward healthy eating. However, the household environment and factors contributing to family chaos may be less recognized within current lessons. Focusing on strengthening order and planning in the food environment of the household could promote a strength-based approach to behavior change within lessons to improve family chaos. The application specifically for households with school-aged children in this review is important because the prevalence of food insecurity is disproportionally high among households with children compared with all US households [1]. The situation of food insecurity within households with children may also introduce varying levels of access or restriction of foods within the household that may be applied to developing educational content. For example, in 7.8% of households with children, only the adults were affected by food insecurity [1]. Parents often protect their child against food security by decreasing their own intake or by saving certain foods for children, yet each household may have varying relationships between members and access to food [1]. Future nutrition education could recognize these complex household dynamics. SNAP-Ed is designed to allow curriculum in each state to tailor educational lessons to the targeted population [78]. Therefore, sensitivity to these issues among target groups of very low food security in the development of educational content may promote a tailored and effective approach to delivery.

The household factors related to dietary quality and food security identified in this review are further applicable to the emerging area of nutrition security [85], the consistent access, availability, and affordability of a diet that promotes health and prevents disease. Nutrition security is equity focused, aligning with an emphasis on tailoring interventions to meet the needs of underserved groups and those with a greater disease burden compared with the general US population. The US Department of Agriculture has defined and set goals to address nutrition security, but a national measure to quantify nutrition security has not yet been developed. Since this review identified factors related to both dietary quality and food security, key aspects of nutrition security, the findings may inform creation of measures and interventions to address nutrition security in households with children. Specifically, components of a measure to assess nutrition security may include queries to some of the household factors identified here that impact dietary quality and food security, such as nutrition knowledge, self-efficacy, attitudes and preferences regarding a healthful diet, barriers to purchasing and preparing healthful foods, and household chaos impacting diet or family meals.

## Limitations

Although the goal of a scoping review is to be broad, exclusion criteria help to narrow the resulting map of the relevant literature for a concise topic with definable boundaries. Limiting the search strategy to only include studies from the past 10 years may have excluded older relevant studies, yet most studies included in the review were published within the past 5 years. The search only included the PubMed database so relevant studies in other databases may not have been included. Additionally, studies that explored exclusively preschool-aged children or younger were not included. These studies may have offered additional relevant findings as some households with young children also include older children and may impact the overall dietary quality and food security in the household. In contrast, this study included all school-aged children, which could range from ages 5 to 19 years, encompassing childhood and adolescent life stages. The household factors identified may vary by the ages within this range and should be considered in application of the findings to particular interventions. This review was limited to household factors contributing to dietary quality and food security in low-income households with school-aged children in the United States and did not explore environmental, community, and social factors that may have an influence on dietary quality and food security in this population. For example, social factors, such as discrimination in policies and practices, could have a myriad of links to the relationships explored in this review and the characteristics of the groups represented. The studies included were observational and most were cross-sectional. This study design is not able to provide support for causal relationships. Several studies included in the review were qualitative, meaning that the findings may have been gleaned from focus groups or interviews and did not necessarily indicate a statistical association with food security or dietary quality. Knowledge of this type should be further evaluated for prevalence in groups and relationships with food security and dietary quality. In addition, the relative importance of the factors summarized is not known. The inclusion of stronger study designs to provide scientific evidence over the long term and randomized experimental evidence would strengthen the links between various themes identified in this review and their potentially causal relationships.

## Conclusion

Parent and child behaviors, food procurement and preparation, and the household environment are household factors that may impact dietary quality and food security in low-income and food-insecure households with school-aged children. These factors are interrelated, and their synergy holds promise for future interventions to improve food security and dietary quality. The findings of this review are especially relevant to inform the design and implementation of nutrition education programming aimed at improving dietary quality and food security in low-income and food-insecure households with children.

## Acknowledgments

The authors acknowledge the contributions of Denise McKeown who helped design the study, completed the search for articles, screened studies for inclusion, integrated the results, and created a discussion that became her thesis, on which this manuscript is based.

### Author contributions

The authors’ responsibilities were as follows – HEM: conceived the study and designed the research plan; BM, HEM: designed the search strategy; HEM, LG: screened the studies for inclusion; HEM: wrote the paper with contributions from LG; HEM: had primary responsibility for final content; and all authors: read and approved the final version of the paper.

### Funding

Supported by the US Department of Agriculture’s National Institute of Food and Agriculture, Agriculture and Food Research Initiative Competitive Grants Program, Foundational and Applied Science Program grant no. 2022-68015-36279/project accession no. 1027912 and US Department of Agriculture Hatch Project (grant 2021-IND90005789). Any opinions, findings, conclusions, or recommendations expressed in this publication are those of the authors and do not necessarily reflect the view of the US Department of Agriculture.

### Author disclosures

HAEM is a member of the *Advances in Nutrition* editorial board and played no role in the Journal’s evaluation of the manuscript. LG, BM, BJM, BAC, WS, and AA report no conflicts of interest.

### Data availability

Data described in the manuscript, code book, and analytic code will be made available upon request pending application and approval.
